# Operating Cooperatively (OC) Sensor for Highly Specific Recognition of Nucleic Acids

**DOI:** 10.1371/journal.pone.0055919

**Published:** 2013-02-18

**Authors:** Evan M. Cornett, Martin R. O’Steen, Dmitry M. Kolpashchikov

**Affiliations:** 1 Chemistry Department, College of Sciences, University of Central Florida, Orlando, Florida, United States of America; 2 Burnett School of Biomedical Sciences, College of Medicine, University of Central Florida, Orlando, Florida, United States of America; Institute of Molecular Genetics IMG-CNR, Italy

## Abstract

Molecular Beacon (MB) probes have been extensively used for nucleic acid analysis because of their ability to produce fluorescent signal in solution instantly after hybridization. The indirect binding of MB probe to a target analyte offers several advantages, including: improved genotyping accuracy and the possibility to analyse folded nucleic acids. Here we report on a new design for MB-based sensor, called ‘*Operating Cooperatively’* (OC), which takes advantage of indirect binding of MB probe to a target analyte. The sensor consists of two unmodified DNA strands, which hybridize to a universal MB probe and a nucleic acid analyte to form a fluorescent complex. OC sensors were designed to analyze two human SNPs and *E.coli* 16S rRNA. High specificity of the approach was demonstrated by the detection of true analyte in over 100 times excess amount of single base substituted analytes. Taking into account the flexibility in the design and the simplicity in optimization, we conclude that OC sensors may become versatile and efficient tools for instant DNA and RNA analysis in homogeneous solution.

## Introduction

**Figure 1:**
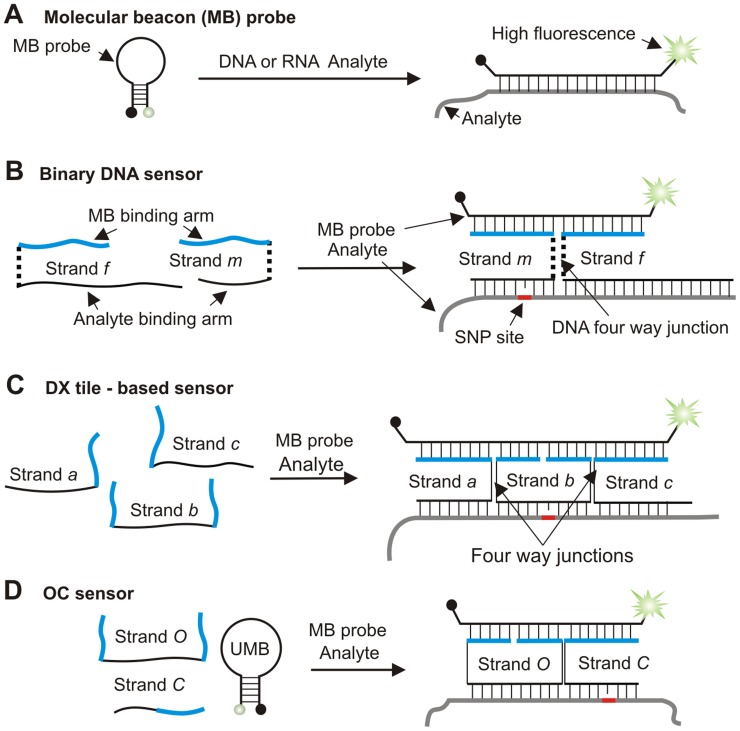
Molecular beacon-based sensors for nucleic acid detection. A) Conventional MB probe [9]. B) MB-based binary DNA sensor (BDS) [32]–[34]. Dashed lines indicate triethylene glycol linkers. C) DX-tile based sensor [35]. D) OC sensor used in this study.

The analysis of single nucleotide variation in DNA and RNA is important for genotyping of single nucleotide polymorphisms [1], [2], somatic mutations [3]–[5] and methylation changes associated with aging and cancer [6]–[8]. A molecular beacon (MB) probe [9] is an extensively utilized hybridization sensor for the analysis of single nucleotide variation [10]–[17]. A traditional MB probe is a stem-loop folded DNA oligonucleotide, with fluorophore and quencher dyes conjugated to the opposite ends (Fig. 1A). MB probes have found multiple applications in the analysis of biological molecules due to their ability to produce a fluorescent signal instantaneously upon hybridization to complementary nucleic acids. MB probes were found to be more specific towards single base substitutions than linear probes [18]–[24].

However, in many cases accurate SNP genotyping still requires elevated temperature and/or measuring melting temperature profiles. Moreover, the design of MB probes remains challenging due to the problems of stem or loop invasion [13], [25], [26] and poor hybridization with structured sequences [27]–[31]. Additionally, MB probes are expensive commercial products ‡ (See note). The use of a limited number of MB probes for the analysis of numerous target sequences is an attractive perspective. In this approach, one or several MB probes can be optimized to avoid stem and loop invasion and used as universal sensors for any DNA or RNA analytes (universal MB probes [32]). This approach may enable multiplex genotyping of millions of SNPs in a low budget format. Here we report a design of a sensor that uses a single MB probe to report multiple nucleic acid sequences. The sensor has a straightforward design and demonstrates excellent SNP specificity at ambient temperatures. Additionally, this new sensor is suitable for the analysis of nucleic acids folded in stable secondary structures, such as bacterial 16S rRNA.

We have been exploring strategies for indirect binding of an MB probe to specific nucleic acids. The first strategy, binary DNA sensors (BDSs), used two DNA adaptor strands (*m* and *f* in Fig. 1B). The *f* and *m* strands hybridized to both an MB probe and an analyte, forming a DNA four way junction-like structure (Fig. 1B). This approach demonstrated unprecedented selectivity and specificity at room temperature [32]–[34]. The BDS had a straightforward design and enabled detection of stem-loop folded sequences [27], [28], [34]. The triethylene glycol linkers, situated between the MB-binding and analyte binding arms (dashed lines in Fig. 1B) were necessary to promote the stabilization of the DNA four-way junction conformation that produces high fluorescence [32], [33]. However, these linkers increased the cost of an adaptor strand by a factor of ∼5 ‡ (See note). In an effort to avoid the non-nucleotide modification we explored alternative approaches, which resulted in the design of a double-crossover (DX) tile sensor (Fig. 1C) [35], [36]. The construct takes advantage of the DX tile, a DNA structure first investigated by Fu and Seeman [37]. The DX tile sensor consisted of an MB probe, three unmodified DNA adaptor strands (*a*, *b*, and *c),* and an analyte, which together formed a pentapartite complex (Fig. 1C). However, the sensor had only moderate selectivity towards a single nucleotide substitution due to the required formation of at least 26 base pairs between the analyte and the adaptor strands. Indeed, long stable hybrids between a probe and a nucleic acid analytes are known to reduce probe selectivity [38]. In an effort to design a nucleic acid sensor that is not cost-prohibitive, yet still maintains excellent SNP selectivity, we report here a new SNP-specific sensor, named ‘*operating cooperatively’* or OC sensor (Fig. 1D). In this study, we demonstrate the exceptional SNP selectivity and specificity of the OC sensor at room temperature, determine the limit of detection (LOD) and demonstrate how this sensor can be tailored to analyze stem-loop folded DNA and bacterial 16S rRNA.

## Materials and Methods

### Reagents

All oligonucleotides (sequences in Table 1) were custom-made by Integrated DNA Technologies, Inc (Coralville, IA). T7 RNA polymerase, PstI, and NTPs were purchased from New England Biolabs (Ipswich, MA). Chemicals were purchased from Sigma-Aldrich (St. Louis, MO). Plasmid pEC16SM was a generous gift from Dr. Dedkova (ASU).

**Table 1:** Oligonucleotides used in the study.

Name	Sequence	Purification
UMB1	5′−/FAM/CGCGT TAAC ATAC AATA GATC GCG/BHQ1/	HPLC
rs14G	5′- ACT GGG CTG ATG TGG GTT CTT TGC AGA ACT GGC TGG CCT CAG AGC AGG GA	SD
rs14A	5′ - ACT GGG CTG ATG TGG GTT CTT TGC AAA ACT GGC TGG CCT CAG AGC AGG GAC CGC GGC CAG TTC TGC AAA GAA CCC ACC GCG G	SD
*E.coli* f-1	T AG TCC GGA TTG GAG TCT GCA ACT CGA CTC CAT GAA GTC GGA AT	SD
*B.sub* f-1	C AG TTC GGA TCG CAG TCT GCA ACT CGA CTG CGT GAA GCT GGA AT	SD
rs87C	5′-ATA CCA CTG CAC TGA AGT ATAAGT ACA TTT TTT GTC ACA CTC TGC TA A CT	SD
rs87T	5′-ATA CCA CTG CAC TGA AGT ATA AGT ATA TTT TTT GTC ACA CTC TGC TAA CT	SD
*O1*	5′-TAT TGT TAT ACT TCA GTG GCG ATC	SD
*C1*	5′-AA TAT ACTATG TTA ACG	SD
*O2*	5′-TAT TGT TCC AAT CCG GAC GCG ATC	SD
*C2*	5′-ATT CCG ACT TCA TGG AGT CGA GTT GCA GAC ATG TTA ACG	SD
*O3*	5′ -TAT TGT AAG AAC CCA CAT GCG ATC	SD
*C3*	5′ -AGT TCT GCA ATG TTA ACG	SD

* Polymorphic sites are underlined.

### Fluorescence Assays

For the fluorescence assays, UMB1 was added to a buffer containing 50 mM MgCl_2_, 50 mM Tris-HCl, pH 7.4, at a final concentration of 50 nM. *O*, *C* strands and either matched (rs87T, *E.coli* f1, rs14G) or mismatched (rs87C, *B.sub* f1, rs14A) strands were added to a final concentration of 200 nM, and 500 nM, respectively. Final sample volumes were 120 µL for DNA analytes and 60 µL for bacterial 16S rRNA analytes. For assays with DNA analytes, samples were incubated for 15 minutes at room temperature (22°C). For fluorescence assays with RNA analytes, samples were incubated for 5 minutes in 90°C water bath, followed by 15 minute incubation at room temperature prior to recording fluorescence spectra. Fluorescence spectra were recorded on a Perkin-Elmer (San Jose, CA) LS-55 Luminescence Spectrometer with a Hamamatsu xenon lamp (excitation at 485 nm; emission 517 nm). All experiments were repeated at least three times, and data is shown as the mean with error bars representing one standard deviation from the mean.

### Preparation Bacterial 16S rRNA Analytes

Total RNA was isolated from *E. coli* (ATCC 8739) and *B. subtilis* (ATCC 9372) using Omega Biotek Bacterial RNA isolation kit following the manufacture’s recommended protocol. Isolated RNA yield was determined, and the presence of 16S rRNA was seen using agarose gel electrophoresis (Fig. S1). *E. coli* 16S rRNA transcript was obtained using in vitro transcription with pEC16SM plasmid, which contains the 16S rRNA gene from *E. coli* strain A19. PstI was used to linearize pEC16SM. The in vitro transcription reaction mixture (300 µL) contained linearized pEC16SM (1 µg), NTPs (4 mM each), T7 RNA polymerase in NEB RNA pol reaction buffer supplemented with MgCl_2_ (20 mM) and bovine serum albumin (25 µg/mL). The reaction was incubated at 37°C for 2 hours. The transcript was collected by ethanol precipitation. The concentration of rRNA transcript was determined by comparison of gel band intensity with gel purified transcript (Fig. S2).

## Results

**Figure 2:**
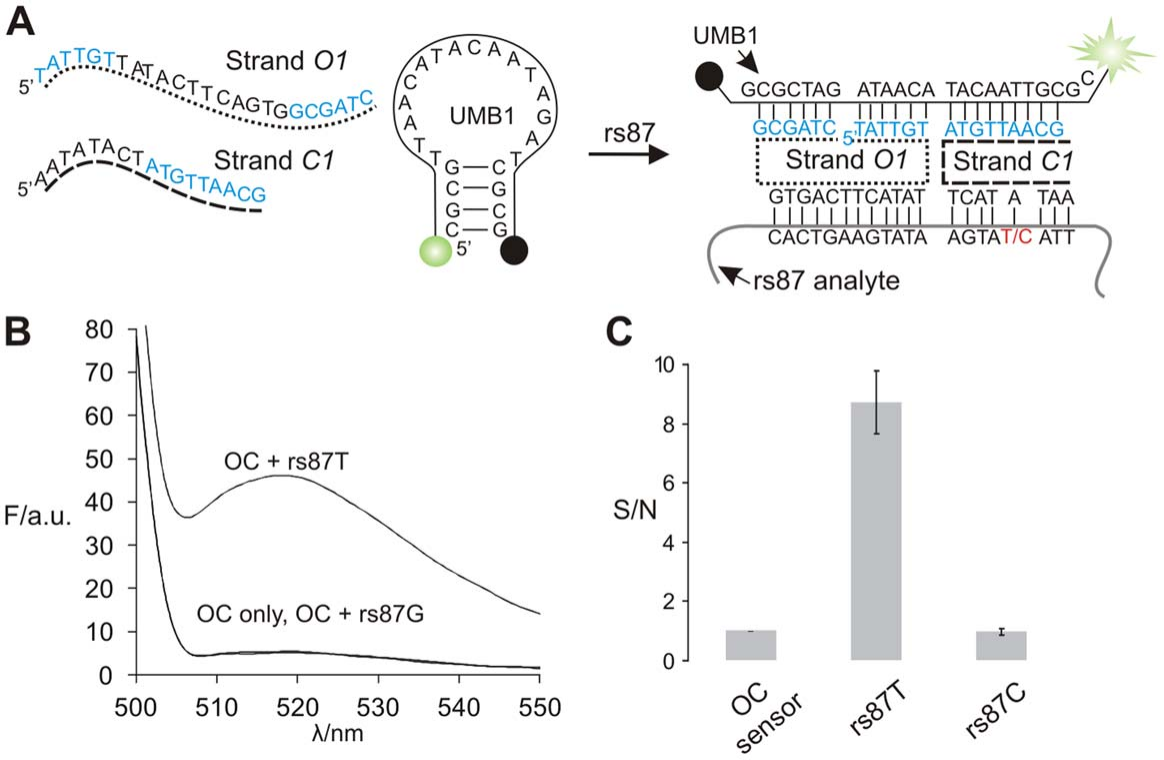
OC sensor for SNP-selective nucleic acid analysis. A) Sensor in complex with rs87T analyte and UMB1 probe. The sensor was designed to recognize the thymidine containing allele, while discriminating against the alternative cytosine containing allele. MB-binding arms are shown in cyan. B) Fluorescent response of OC sensor specific to rs87T allele sequence in the absence or presence of specific or non-specific analytes. C) Signal-to-noise ratios of four independent experiments with error bars indicating one standard deviation from the mean. The complete sequences of all oligonucleotides are included in Table 1.

**Figure 3:**
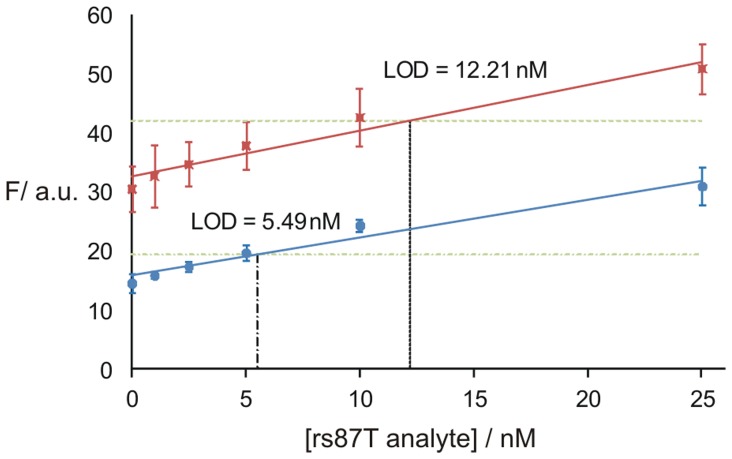
Limit of detection (LOD) for the OC sensor. *O1, C1,* and UMB1 probe were used to analyze low concentrations of the matched analyte (rs87T). The limit of detection (LOD) was calculated as the analyte concentration that triggered a fluorescent signal equal to the average fluorescence of the background from three independent measurements plus three standard deviations of the average background fluorescence. Data shown were plotted as the mean with error bars representing one standard deviation from the mean from three independent trials. LOD was determined in the absence (blue line) or in the presence (red line) of 500 nM of mismatched analyte (rs87C). The green dashed lines represent the respective thresholds and the black dashed lines represent the respective LODs.

**Figure 4:**
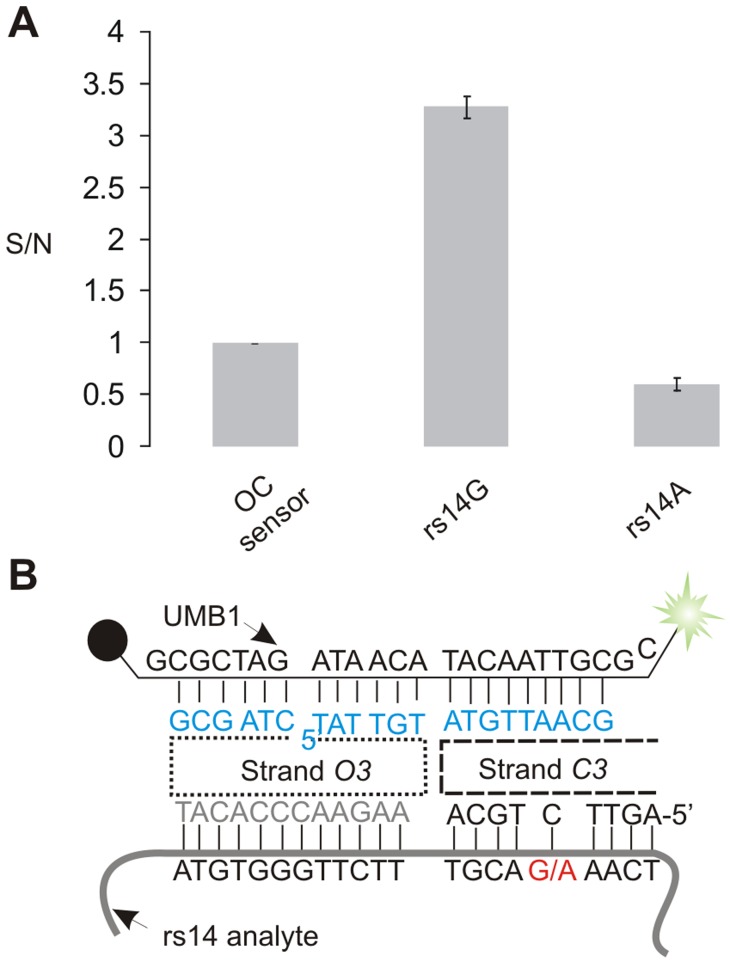
OC sensor designed to detect rs14 human SNP. A) Signal to noise ratios for OC sensor. B) The OC sensor complex formed with UMB1 probe and rs14G analyte.

The OC sensor uses two unmodified DNA adaptor strands (*O* and *C),* each of which binds to a MB probe and an analyte (Fig. 1D). The design of the sensor was adjusted to room temperature (22°C). For these conditions, strand *O* had two 5 or 6 nucleotide MB- binding arms. Thus, the two short binding arms had a very weak interaction with MB probe in the absence of analyte, thus ensuring low fluorescent background. Strand *C* had 8 or 9 nucleotide MB binding arms; this length was required for the formation of stable DNA four-way junction associates [37]. The analyte binding arm of strand C can be adjusted to provide high mismatch selectivity or stabilization of the OC complex as demonstrated below. Figure 2A shows a design of the OC sensor for the recognition of a human genomic DNA sequence that is known to have a SNP. This analyte contains a mutation which has been suggested as a useful marker for human identification in forensic applications [39]. The sensor fluoresced only in the presence of the matched rs87T sequence, but not in the presence of an alternative allele (Fig. 2B). The limit of detection (LOD) of the sensor was found to be ∼5.5 nM (Fig. 3 blue line), which is somewhat above the detection limit of a typical MB probe [9]–[13], [32]. Importantly, the presence of high excess (500 nM) of a single-based substituted analyte did not jeopardize the assay: the cognate analyte was detected with the LOD of ∼12 nM (Fig. 3 red line). Importantly, the LOD is well below the concentration achievable by PCR (∼100 nM). In order to demonstrate general applicability of the approach, an OC sensor was designed to recognize an alternative human SNP (rs14 in Fig. 4). Again, the sensor demonstrated excellent SNP discrimination at room temperature. The design of the new sensor was straightforward: (i) the sensor utilized the same UMB probe; (ii) only the analyte-binding arms of the sensor were changed to complement to the new target.

**Figure 5:**
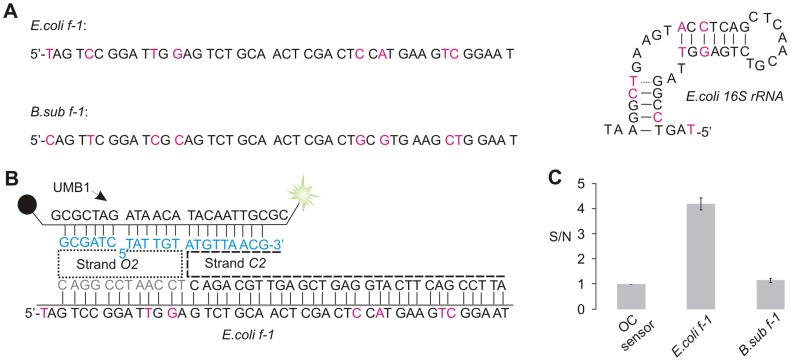
OC sensor for detection of secondary structure-forming fragment of *E.coli* 16S rRNA. A) Sequences and the predicted secondary structure of stem-loop folded fragments of 16S rRNA. The nucleotide variations in *E. coli* and *B. subtilis* 16S rRNA are shown in magenta. B) OC sensor in the complex with fully matched *E.coli f-1* sequence. MB binding arms of strands *O2* and *C2* are shown in cyan. C) Signal-to-noise ratio of fluorescent response of OC sensor in the presence of absence of *E.coli* or *B. subtilis* 16S rRNA mimics. The assay was conducted in 50 mM Tris HCl, pH 7.4, 50 mM MgCl_2_ at room temperature (22°C). The data represents the signal to noise ratios of three independent experiments with error bars indicating one standard deviation from the mean.

Natural single stranded nucleic acids are often folded in stable secondary structures. Stable structures prevent hybridization probes, including the MB probe, from interacting with such analytes [27]–[31], [40]. A common approach to overcome this difficulty is to target only unstructured fragments of natural RNAs. This limits the choice of hybridization probes and often prevents analysis of SNPs located in stem regions of such analytes. Here we demonstrate that OC sensor is capable of analysing folded nucleic acids. As a model, a fragment of *E. coli* 16S rRNA (*E. coli f-1* in Fig. 5A) was used as a specific analyte and a corresponding fragment of *B. subtilis* 16S rRNA (*B. sub f-1* in Figure 3A) was used as a non-specific analyte. These analytes were DNA olignonucleotide mimics of actual RNA sequence. Analysis of 16S rRNA is important for classification of bacteria species and for molecular diagnostics of infectious diseases [41]–[44]. On the other hand, 16S rRNAs, as well as their DNA amplicons can fail to hybridize to oligonucleotide probes [45], [46]. In this study we have randomly selected a fragment of *E. coli* 16S rRNA folded in a typical stem-loop structure with a bulge in the middle of the stem (Fig. 5A). The predicted melting temperature of this stem was 84.5°C according to IDT Oligo Analyser software (experimental conditions set at 50 mM MgCl_2_, 50 mM monocharged ion).

OC sensor capable of analysing stem-loop folded *E.coli f-1* took advantage of *C2* strand equipped with a long analyte binding arm, containing 30 nucleotides (Fig. 5B). The long arm allows the *C2* strand to tightly hybridize to an extended portion of the analyte and unwind its secondary structure. However, the long arm may also allow a less stable complex to form between *C2* and *B.sub f-1* analyte. Thus, the *O2* strand is designed to contain an analyte binding arm of only 12 nucleotides and will hybridize tightly only to the fully complementary *E.coli f1*. Indeed, high fluorescence was registered only in the presence of a fully matched strand, but not in the presence of a mismatched *B.sub f-1* (Fig. 5C).

**Figure 6:**
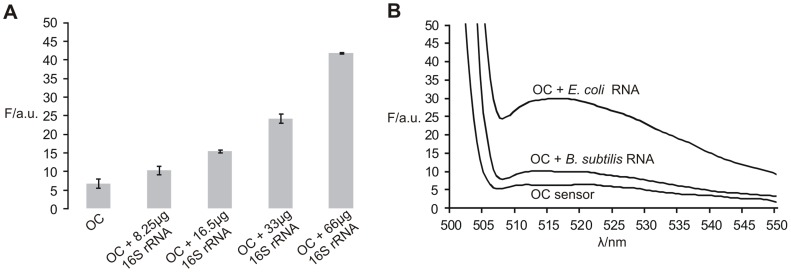
Detection of bacterial 16S rRNA with OC sensor. A) Signal-to-noise ratios for OC sensor detection of various concentrations of *E. coli* 16S rRNA in vitro transcript. Average signal-to-noise (S/N) values of three independent measurements with a standard deviation are presented. B) Fluorescent spectra of OC sensor detection of 16S rRNA from bacteria total RNA isolation. The assays were conducted in 50 mM Tris HCl, pH 7.4, 50 mM MgCl_2_ with a 5 minute annealing step (90°C) followed by 15 minute incubation at room temperature (22°C).

In order to test the OC sensor against actual bacterial 16S rRNA samples, we obtained of *E. coli* 16S rRNA by in vitro transcript. The OC sensor was able to recognize and give high fluorescent signal in the presence of the 16S rRNA transcript (Fig. 6A). However, an additional annealing step was required, likely due to a very stable structure of the long 16S rRNA compared to our mimic analytes. Additionally, the OC sensor was applied to the identification of 16S rRNA collected from bacteria samples. The total RNA from *E. coli* and *B. subtilis* was obtained and the OC sensor correctly identified the *E. coli* 16S rRNA. The OC sensor produced a high fluorescence signal in the presence of *E. coli* RNA, but gave a low signal in the presence of *B. subtilis* RNA or no analyte (Fig. 6B). Thus, the OC sensor is suitable for sequence specific detection of conformational constrained natural RNAs.

## Discussion

MB probes have become an important tool of molecular diagnostics, especially in real-time PCR (rtPCR) format [10], [12]. However, the application of MB probes in practice suffers from a number of problems including stem invasion, and inability to analyse folded RNA and DNA. Moreover, despite improved selectivity towards mismatched analytes, MB probes still require monitoring of melting profiles for accurate SNP genotyping [9]–[12]. Multicomponent sensors that use indirect binding of an MB probe to a target DNA or RNA analyte may significantly contribute to a solution for these pitfalls. In this approach a limited number of optimized MB probes, *universal molecular beacon probes*, can provide a toolbox for the analysis of millions of human and bacterial SNPs with no need to design a pair of unique MB probes for each new SNP of interest.

We have previously introduced two different designs for MB probe-based multicomponent sensors, each with its respective disadvantages [33], [36]. However, the OC sensor demonstrated marked improvement over the previous designs: it is less expensive than binary DNA sensor and more selective towards SNPs than a DX-tile sensor. Additionally, the OC sensor is suitable for the analysis of secondary structure folded DNA and RNA. Herein, the utility of the OC sensor was demonstrated by adapting an OC sensor for detection of bacterial 16S rRNA. Therefore, the approach described in this study has a potential to become useful for instantaneous detection of nucleic acids in homogeneous solutions. OC sensors used in this study were designed to operate at room temperature to avoid temperature control for hybridization reaction. However, the design can be also adjusted to elevated temperatures (50–60°C) if the probes to be used in quantitative real time PCR (data not shown).

### Conclusion

OC sensor produces fluorescent signal instantaneously upon hybridization to a specific nucleic analyte. MB probe binds to the analyte indirectly, e.g. through two unmodified DNA oligonucleotide adaptor strands. This gives an opportunity to optimize a single MB probe for the analysis of multiple DNA or RNA sequences. The approach provides a versatile tool for nucleic acid analysis at ambient temperature that is SNP specific and capable of analysing stem-loop folded nucleic acids. Thus the OC sensor represents a promising tool for SNP analysis in homogeneous solution, which might be used for detection and genotyping of bacteria or human SNPs.

### Notes

Synthesis of a MB probe (10 nmol minimum garanteed yeild) by Integated DNA technology cost $405.00–780.00, while the synthesis of both strands of OC probe (20 nmol minimim garanteed yeild) costs<$15.00. A TEG linker-containing DNA strand cost ∼$ 50.00 (100 nmol synthetic scale).

## Supporting Information

Figure S1 Agarose analysis of total RNA isolation from *E. coli* and *B. subtilis.*
(TIF)

Figure S2 In vitro *E. coli* 16S rRNA transcript concentration determination by 1% agarose gel electrophoresis with GELRED staining. Transcript was gel purified and concentration determined using a spectrophotometer. Several concentrations of known gel purified transcript (lanes 1–4), as well as 1 µL from two dilutions of unknown concentration used in the fluorescent assays. The 1/8 dilution sample was determined to be equal in intensity to 166 ng band. This was used to determine the concentration of all in vitro transcript samples used in fluorescent assays.
(TIF)
