# Supplementary figures and images for: Operating Cooperatively (OC) Sensor for Highly Specific Recognition of Nucleic Acids
Source: PLoS One. 2013 Feb 18;8(2):e55919. doi: 10.1371/journal.pone.0055919 (PMC3575382; doi:10.1371/journal.pone.0055919)

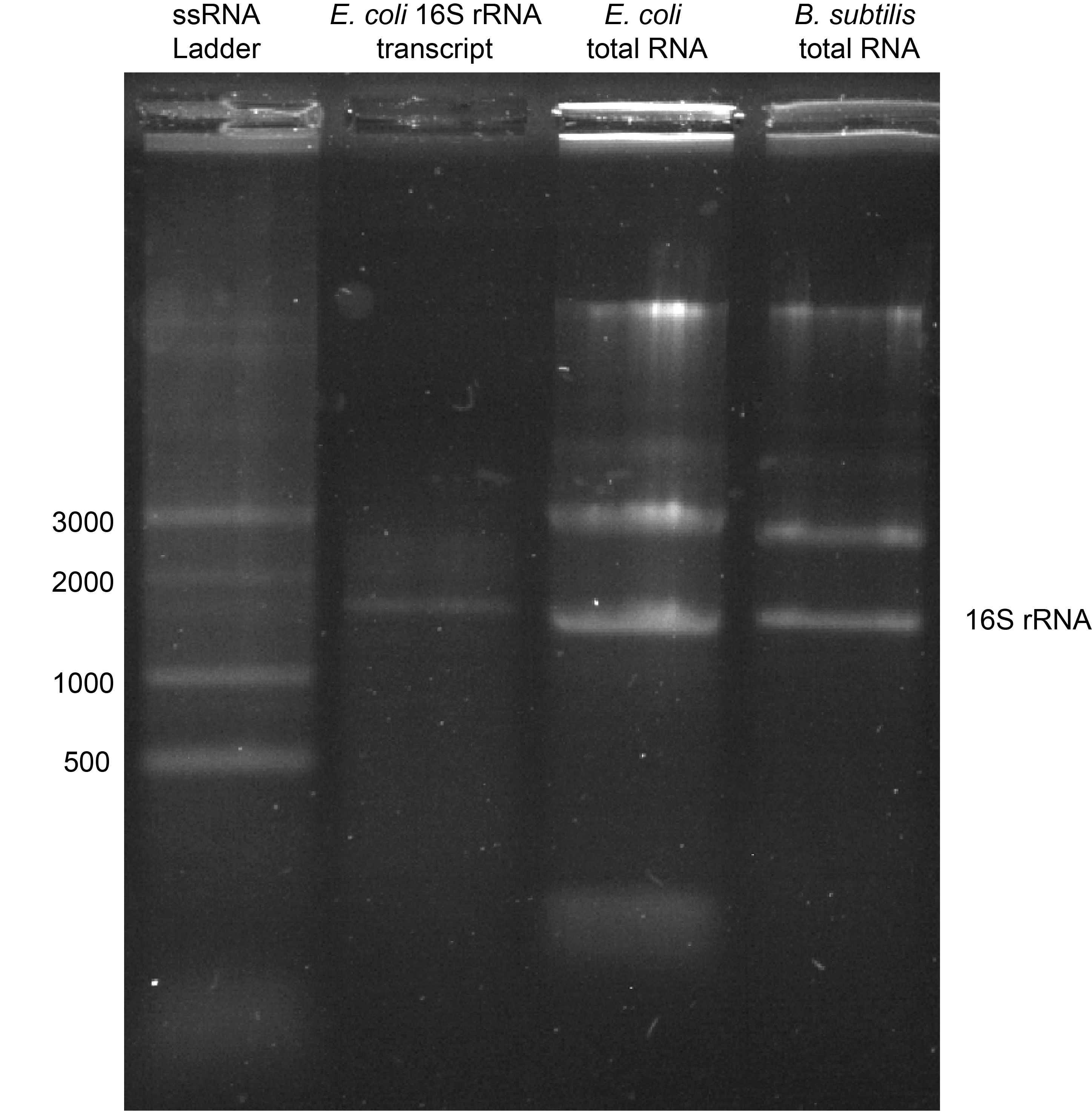

Supplement: Figure S1 — Agarose analysis of total RNA isolation from E. coli and B. subtilis. (TIF) [file pone.0055919.s001.tif]

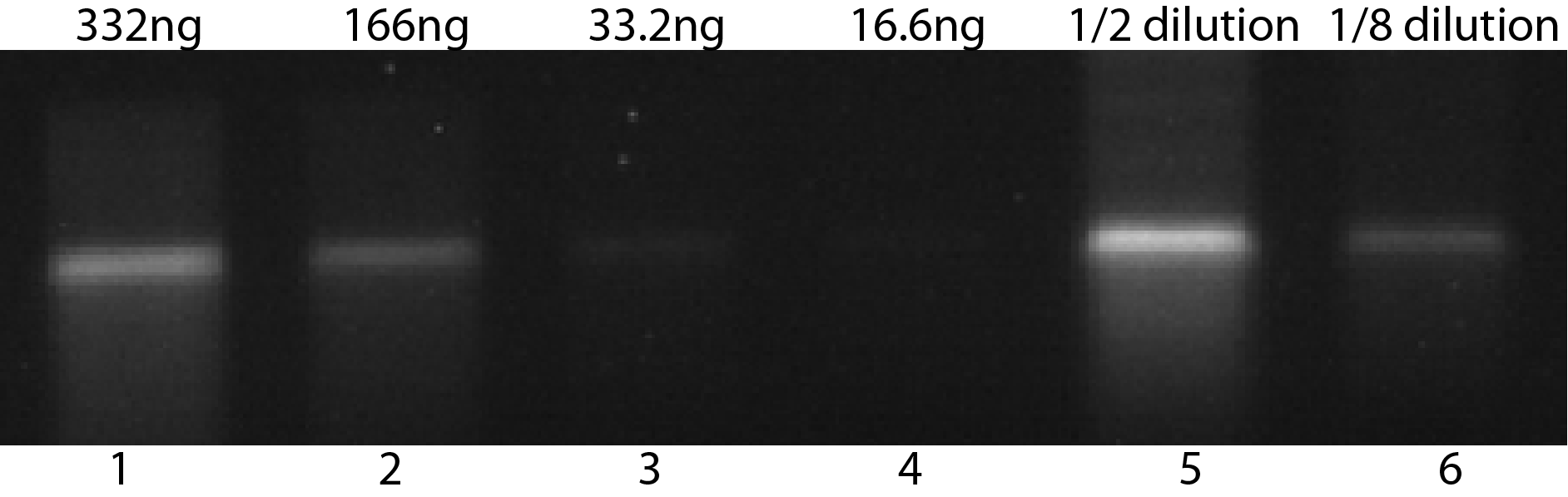

Supplement: Figure S2 — In vitro E. coli 16S rRNA transcript concentration determination by 1% agarose gel electrophoresis with GELRED staining. Transcript was gel purified and concentration determined using a spectrophotometer. Several concentrations of known gel purified transcript (lanes 1–4), as well as 1 µL from two dilutions of unknown concentration used in the fluorescent assays. The 1/8 dilution sample was determined to be equal in intensity to 166 ng band. This was used to determine the concentration of all in vitro transcript samples used in fluorescent assays. (TIF) [file pone.0055919.s002.tif]
